# Transcriptome Analysis Reveals Dynamic Fat Accumulation in the Walnut Kernel

**DOI:** 10.1155/2018/8931651

**Published:** 2018-11-28

**Authors:** Shuang Zhao, Xuemei Zhang, Yanping Su, Yilan Chen, Yang Liu, Meng Sun, Guohui Qi

**Affiliations:** ^1^Department of Forestry, Agricultural University of Hebei, Baoding 071000, China; ^2^Cotton Research Institute, Hebei Academy of Agriculture and Forestry Sciences, Shijiazhuang 050000, China; ^3^Research Center for Walnut Engineering and Technology of Hebei, Lincheng 054304, China; ^4^Department of Life Science, Normal University of Langfang, Langfang 065000, China; ^5^Baoding Forest Seedling Management Station, Baoding 071000, China

## Abstract

Walnut (*Juglans regia* L.) is an important woody oilseed species cultivated throughout the world. In this study, comparative transcript profiling was performed using high-throughput RNA sequencing technology at the following three stages of walnut fat synthesis in the “Lvling” walnut cultivar: the initial developmental stage (L1), the fast developing stage (L2), and the last developing stage (L3). A total of 68.18 GB of data were obtained on the three developmental stages, and 92% to 94% of clean data were able to be located to the reference genome. Further comparisons of the transcripts in the three libraries revealed that 724, 2027, and 4817 genes were differentially expressed between the L2 and L1 (L2vsL1), L3 and L2 (L3vsL2), and L3 and L1 (L3vsL1) samples, respectively. Through the GO gene enrichment analysis, differentially expressed genes (DEGs) in L2vsL1, L3vsL2, and L3vsL1 were enriched into 3, 0, and 2 functional categories, respectively. According to the KEGG enrichment analysis, DEGs in L2vsL1, L3vsL2, and L3vsL1 were annotated into 77, 110, and 3717 taxonomic metabolic pathways in the KEGG database, respectively. Next, we analyzed expression levels of genes related to fat synthesis. Our results indicated that ACCase, LACS, and FAD7 were the key genes related to fat synthesis. The high-throughput transcriptome sequencing of walnut in different developmental stages has greatly enriched the current genomic available resources. The comparison of DEGs under different developmental stages identified a wealth of candidate genes involved in fat synthesis, which will facilitate further genetic improvement and molecular studies of the walnut.

## 1. Introduction

Edible oil, an important source of fat for humans, has received increasing attention as a necessary dietary ingredient. However, China edible oil is highly dependent on foreign imports. In 2013, a total of 11.694 million tons of oil were extruded from Chinese-produced oilseed crops, but the annual total demand for edible oil was 30.408 million tons; therefore, the self-sufficiency rate for edible oil in China was 38.5% [1]. The dependence on foreign edible oil increased to 61.5%, far exceeding the international security warning line. It is projected that by 2030, the demand for vegetable oil will double. This demand can be met only by increasing the oil content of current oilseed crops or introducing new high-yield oil crops [2].

Walnut (*Juglans regia* L.) is an important woody oilseed species. Walnut oil, a high-quality cooking oil, is comparable to olive oil [3]. The fat content of walnut can be as high as 65.21 g/100 g, and polyunsaturated fatty acids account for 47.17 g/100 g; the polyunsaturated fatty acids are primarily linoleic acid (38.90 g/100 g) and linolenic acid (9.08 g/100 g). The ratio of oleic acid (*ω*-6) to *α*-linolenic acid (*ω*-3) in walnut kernels is close to (4–6) : 1, and this nutritional component is beneficial for human health. This component can effectively prevent cardiovascular disease and is beneficial for type 2 diabetes patients [4, 5]. Given that walnut oil is an important functional plant fat, this oil is being widely studied in various countries. Increasing walnut yields and selecting high-oil walnut varieties are essential strategies for increasing the production of high-grade edible oil and reducing China's dependence on imported edible oil. Understanding the genetic mechanism of walnut oil metabolism regulation is important to improve the oil content of walnut and provide an important theoretical basis for research.

The transcriptome is the total expressed RNA of a specific tissue or cell at a certain stage of development or in a specific state. Transcriptome analysis can reveal differences in the expression level of the same gene in different states and can explore the interaction between different genes and the functional characteristics of each gene [6]. Transcriptome analysis can obtain information on gene expression levels, structural characteristics, function, and intergene interactions; therefore, this technique is an effective means to study specific biological processes and molecular mechanisms under biological conditions [7]. In recent years, many scholars have used transcriptome sequencing to research fat metabolism in many species, such as oil palm [8, 9], castor [10], *Jatropha curcas* [11, 12], *Camellia oleifera* [13], *Idesia polycarpa* [14], and *Symplocos paniculata* [15]. The sequencing and transcriptome analyses of oil palm revealed that *WRINKLED1 (WRI1)* regulates the accumulation of pericarp fat in oil palm, but the expression of *WRI1* is 75 times higher in the pericarp than in the seed; *WRI1* plays a central role in nut fat biosynthesis. *LEAFY COTYLEDON1 (LEC1)*, *LEC2*, *ABSCISIC ACID INSENSITIVE3 (ABI3)*, and *FUSCA3* are expressed in nuts but not in the mesocarp. *PICKLE (PKL)*, a transcriptional regulator, is expressed in both the mesocarp and the kernel. Research on *Idesia polycarpa* by Li et al. [14] also revealed that the expression of the oil-related transcription factor WRI1 is higher in the pulp than in the seeds, whereas other transcription factors, *LEC1*, *LEC2*, *ABI4*, *ABI3*, and *FUS3*, are expressed only in the seeds. An analysis of acetyl-CoA activity in castor by Brown et al. [10] revealed that ricinoleic acid acyl-CoA does not dominate during endosperm oil formation. This finding suggested that the expression of *RcDGAT2* and *RcPDAT1* increased oleate-12 hydroxylase activity, thereby increasing the ricinoleic acid content of the organ. Liu et al. [15] analyzed the transcriptome of *S. paniculata* at different development stages; the result founded that the key regulatory enzymes involved in lipid metabolism *(ACC*, *KASII*, *KASIII*, *FATA*, *FATB*, *ACSL*, *SAD*, *FAD2*, *GPAT9*, *LPCAT1*, *DGAT1*, and *PDAT1)* were determined and they play vital roles in the oil accumulation in *S. paniculata* fruit and fatty acid composition of the fruit oil.

In this study, transcriptome sequencing technology was used to analyze differentially expressed genes of the walnut kernels at various developmental stages and to explore the mechanism underlying walnut fat synthesis at the gene transcriptional level. The transcriptional expression information of specific genes during the development of the walnut was analyzed. This information provides a theoretical reference for future research on the mechanism underlying the synthesis of the fat in the walnut kernel, laying a foundation for the cloning and functional analysis of genes related to oil synthesis.

## 2. Materials and Methods

### 2.1. Plant Materials

The walnut cultivar Lvling was used in this study. Three 7-year-old trees that were disease-free and exhibited consistent, vigorous production were selected. Beginning at 50 days after fertilization (DAF), samples were taken every 10 days, ending at 130 DAF. A total of 5 fruits with normal growth, no disease and consistent size were collected in each direction (north, south, west, and east) on each tree canopy, that is, 20 fruits were collected per plant. The samples used for the transcriptome sequencing were the fruits collected at 60, 90, and 120 DAF. The kernels were removed and put into self-sealing bags, labeled as samples L1 (60 DAF), L2 (90 DAF), and L3 (120 DAF), with three biological replicates each. The samples were rapidly frozen with liquid nitrogen and stored in a −80°C freezer. The remaining fruits, which were used for the analysis of fat and fatty acid content, were removed from the husk and placed in an oven and dried at 40°C until their weight stabilized.

### 2.2. Determination of Fat and Fatty Acid Content

The crude fat content was measured by Soxhlet extraction [16], and then the fat was methyl esterified. The methyl esterified fatty acid mixture was analyzed using gas chromatography [17, 18], and the peak area normalization method was used to calculate the components in a unified way. There were three replicates in each treatment, and each experiment was repeated three times. Data were analyzed using the SPSS software (version 17). Significant differences were assessed by using Duncan's multiple range test at the 5% probability level. All the data were expressed as mean ± standard error (SE).

The formula (*M*_*n*+1_ − *M*_*n*_) represents the increase of kernel fat in two consecutive samplings, the formula (*T*_*n*+1_ − *T*_*n*_) represents the number of days between two consecutive samplings, and the formula for calculating the walnut kernel fat accumulation rate is as follows: KW (g d^−1^) = (*M*_*n*+1_ − *M*_*n*_)/(*T*_*n*+1_ − *T*_*n*_).

### 2.3. RNA Extraction and Quality Verification

RNA extraction from samples and transcriptome sequencing was completed by Beijing Novo Gene Technology Co. Ltd. The total RNA of the walnut kernel samples was extracted using the Tiangen DP441 Polysaccharide Polyphenol Kit. The purity, concentration, and integrity of the RNA samples were accurately detected using 1% agarose gel electrophoresis, NanoDrop 2000, Qubit 2.0, and Agilent 2100.

### 2.4. cDNA Library Construction and Sequencing

After the samples were qualified, the eukaryotic mRNA was enriched using magnetic beads with oligo (dT) bound to the polyA tail of the mRNA by A-T complementary pairing. The mRNA was then broken into short fragments, and single-stranded cDNA was synthesized with a six-base random primer using mRNA as a template. Then, double-stranded cDNA was synthesized by adding buffer solution, dNTPs, and DNA polymerase I, followed by the purification of the double-stranded cDNA using AMPure XP beads. The purified double-stranded cDNA was then subjected to end repair, an A tail was added, and the sequencing adapter was ligated. Next, the fragment size was selected by AMPure XP beads, and PCR enrichment was performed to obtain the final cDNA library. After the construction of the library, the length of the inserted fragments in the library was detected using the Agilent 2100, and the effective concentration of the library was accurately quantified (effective concentration of the library: >2 nM) by qPCR to ensure library quality. After passing the library check, Illumina HiSeq sequencing was performed after pooling the libraries according to the requirements of effective concentration and target data volume.

### 2.5. Raw Sequencing Data Processing and Sequence Assembly

The raw image data files obtained by high-throughput sequencing were converted into the sequenced reads by raw CASAVA (base calling) analysis, namely, raw reads. To obtain clean reads, the raw reads were filtered to remove the reads with adapters, the reads of *N*, which accounted for more than 10% of the reads (*N* indicates that the base information cannot be determined) and the low-quality reads (*Q* phred ≤ 20 bases for the entire length of the read of the 50% above reads). The follow-up analyses were all based on the resulting clean reads. The HISAT software was selected to analyze the genome localization on the filtered sequencing sequence; the reference genome path is https://treegenesdb.org/Drupal/FTP/Genomes/Reju/v1.0/.

### 2.6. Screening and Analysis of Differentially Expressed Genes

After DESeq standardization, differential expression analysis was performed. The standard of differential gene screening was padj < 0.05. A gene ontology (GO) functional analysis and a KEGG pathway enrichment analysis were used to determine differentially expressed genes.

### 2.7. Real-Time qRT-PCR Analysis

Eight unigenes involved in walnut fat accumulation were chosen for validation by qRT-PCR. In this experiment, the reference gene was *β*-actin. The primers were designed with the Primer Premier 6.0 software (Table 1). RNA was extracted from the fruit according to the previously detailed method. The qRT-PCR was performed using an Ultra SYBR Mixture (With ROX) (CWbio Co. Ltd, cat no. CW0956) on Line Gene 9600 Plus Fluorescence quantitative PCR apparatus (Bioer, Hangzhou, China). Three replicates of each sample were conducted to calculate the average Ct values. The relative expression level was calculated by the comparative 2^−ΔΔCt^ method [19, 20].

## 3. Results

### 3.1. Changes of Fat Content in the Walnut Fruit at Various Developmental Stages

The walnut embryos were in the sol state at the early stage of development and began to solidify at 50 DAF, at which time the walnut kernel fat content was 8.3%. The rate of kernel fat at 50 to 60 DAF increased was low, whereas the period at 60 to 90 DAF exhibited the fastest increase. At 90 to 120 DAF, the increase of fat content slowed down. At 120 to 130 DAF, the walnut was completely mature, and the fat content tended to stop increasing. The fat content at 130 DAF was 69.8%.

**Figure 1:**
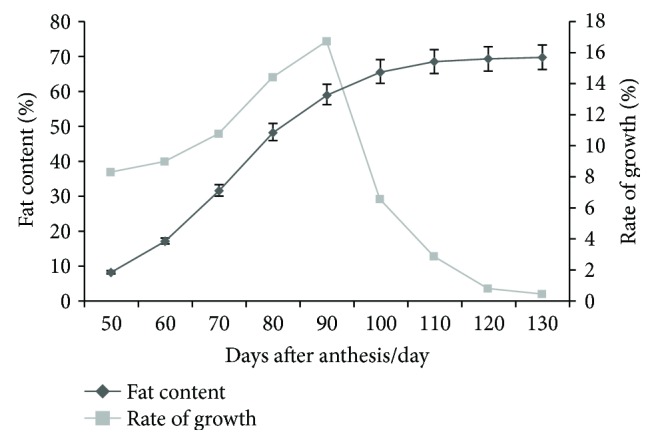
Dynamic changes of fat content in walnut kernel.

The accumulation rate of walnut fat displays a single peak curve (Figure 1). The fat slowly accumulated between 0 and 60 DAF and accumulated rapidly at 60 to 80 DAF, then reaching a peak at 90 DAF. Thereafter, the fat accumulated slowly until 130 DAF, at which point the fruits were fully mature and the fat content tended to stop increasing.

Based on the analysis of the kernel fat content at various developmental stages of Lvling walnuts, the optimal periods for analyzing transcriptomics were determined to be the early stage of fat synthesis (60 DAF), the peak stage of fat synthesis (90 DAF), and the late stage of fat synthesis (120 DAF).

### 3.2. Changes of Fatty Acid Content in the Walnut Kernel at Various Developmental Stages

A total of 14 kinds fatty acids have been detected in the walnut kernel, including 6 unsaturated fatty acids and 8 saturated fatty acids (Table 2). Walnut fat was composed primarily of the unsaturated fatty acids (linoleic acid, oleic acid, and *α*-linolenic acid) and the saturated fatty acids (palmitic acid and stearic acid). The total relative content of these five fatty acids was found to be greater than 98% at each period. The linoleic acid content gradually increased from 60 days to 90 DAF, reaching the highest value of 69.66% at 90 DAF and then gradually decreasing, reaching 63.29% at 120 DAF. Oleic acid showed an upward trend from 60 to 120 DAF, reaching 20.15% at 120 DAF and then dropping by 0.87% at 130 DAF. *α*-Linolenic acid showed an upward trend at 60 to 70 DAF, reaching a maximum of 12.22% at 70 DAF and then declining. At 90 DAF, the *α*-linolenic acid content slightly increased and then exhibited a downward trend. The content of palmitic acid was 17.45% at 60 DAF, reaching the lowest value at 100 DAF and then gradually increasing over the subsequent month to reach 6.46% at 130 DAF. The content of stearic acid was relatively smaller, reached the highest value, 2.87%, at 100 DAF. The lowest palmitic acid content observed was 2.55% at 130 DAF. Based on the above analysis, the relative content of saturated fatty acids and unsaturated fatty acids in walnut kernel fats displayed opposing trends: the saturated fatty acids gradually declined, whereas the unsaturated fatty acids showed a gradual upward trend.

#### 3.2.1. Transcriptome Sequencing Data Analysis

#### 3.2.2. Transcriptome Sequencing Quality

A total of 4.73 × 10^8^ raw reads were obtained after sequencing the transcriptome from three developmental stages, and 4.54 × 10^8^ clean reads remained after filtration (Table 3). A total of 68.18 GB of data were obtained. The Q20 of clean reads were all above 96.75%, the percentage of Q30 bases was between 91.88% and 92.63%, and the GC content was approximately 46.49% to 49.64%. The square of the Pearson's correlation coefficient (*R*^2^) between each sample repetition was greater than 0.8 in every case, indicating that the repeatability among the samples was satisfactory and could be used for subsequent bioinformatics analysis.

#### 3.2.3. Comparison of Transcriptome Data with Reference Sequences

**Figure 2:**
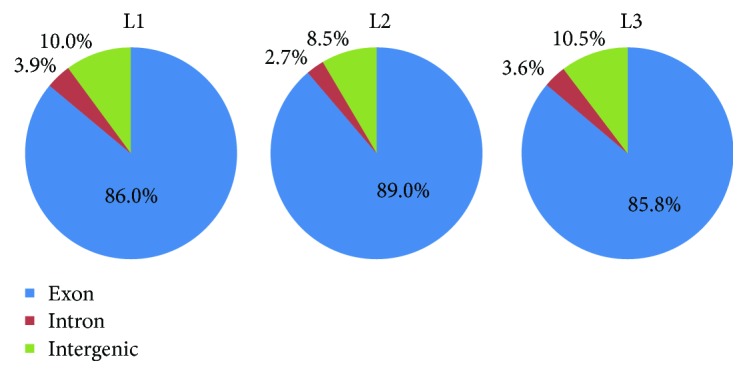
Distribution of reads in different regions of the genome in three periods.

The alignment results showed that between 92% and 94% of the clean data could be located on the genomes, and the percentage of unique alignment positions was above 74% (Figure 2). Over 85% of the sequences aligned to the genome were located in exon regions. A small percentage of the sequences were found to be located in intergenic regions, likely due to the incomplete annotation of the genome, and a small percentage of the sequences were located in intron regions, which may be due to the retention of mRNA precursors and the inactivation of variable cleavage.

#### 3.2.4. Analysis of Differentially Expressed Genes

**Figure 3:**
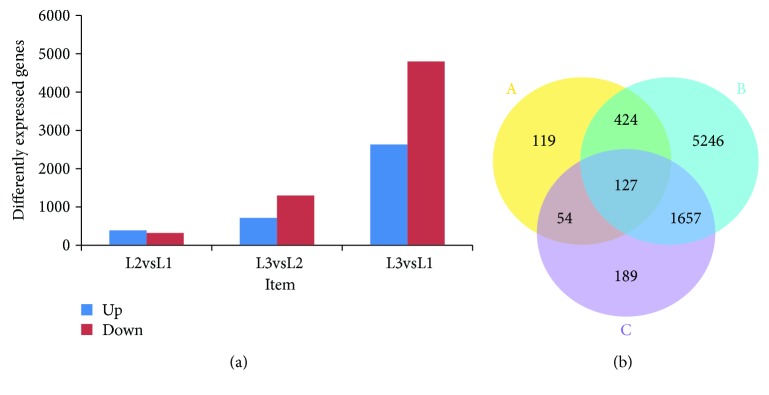
Distribution of differentially expressed genes in walnut kernel at different developmental stages. (a) Number of differentially expressed genes in different combinations. (b) Venn diagram of differentially expressed genes in different combinations: A: L2vsL1; B: L3vsL1; and C: L3vsL2.

*(1) Distribution of Differentially Expressed Genes in the Walnut Kernel at Various Developmental Stages*. The results of the analysis of differentially expressed genes in the walnut kernel at various developmental stages showed that the number of differentially expressed genes in L2vsL1, L3vsL2, and L3vsL1 was 724, 2027, and 4817, respectively (Figure 3(a)). The corresponding number of upregulated and downregulated genes was 400, 724, and 2637 and 324, 1303, and 4817, respectively. The number of upregulated genes was higher than that of downregulated genes in the case of L2vsL1, but the number of downregulated genes in the other two comparisons was significantly higher than that of upregulated genes.

As shown in the Wien diagram of differentially expressed genes in different combinations (Figure 3(b)), the specific DEGs of L2vsL1, L3vsL1, and L3vsL2 were 119, 5246, and 189, respectively. There were 424, 1657, and 54 genes that were differentially expressed simultaneously in two combinations, and 127 genes were differentially expressed across all three combinations. Among them, 54 genes were related to metabolism, and 33 were related to the synthesis of secondary metabolites (Table 4).

**Figure 4:**
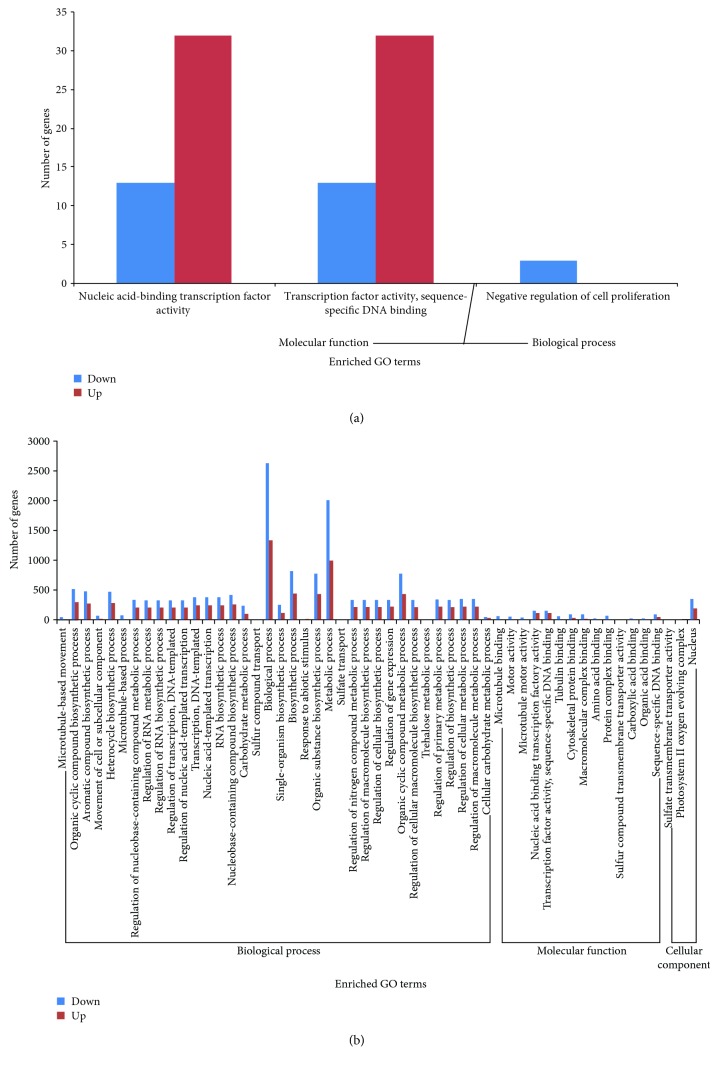
Clustering analysis diagram of significantly enriched GO terms from differentially expressed genes between different samples. (a) Clustering analysis diagram of significantly enriched GO terms from differentially expressed genes in L2vsL1. (b) Clustering analysis diagram of significantly enriched GO terms from differentially expressed genes in L3vsL1.

*(2) GO Enrichment Analysis of Differentially Expressed Genes*. A GO functional enrichment analysis of differentially expressed genes in the walnut kernel at various developmental stages was conducted. As shown in Figure 4, the genes expressed in L3vsL2 were not significantly enriched, whereas the genes differentially expressed in L2vsL1 were significantly enriched in the following functional categories (Figure 4(a)): nucleic acid-binding transcription factor activity, transcription factor activity, sequence-specific DNA binding, and negative regulation of cell proliferation. The genes differentially expressed in L3vsL1 were enriched predominantly in the following two major categories: biological processes and molecular functions (Figure 4(b)). In the biological processes group, biochemical processes and metabolic processes accounted for the highest proportion, followed by the biosynthesis of each substance, nucleic acid template transcription, DNA template transcription, macromolecular metabolic process regulation, and gene expression regulation. In the molecular functional group, sequence-specific DNA synthesis transcription factor activity and nucleic acid-binding transcription factor activity accounted for the highest proportion, followed by sequence-specific DNA synthesis, cytoskeletal protein synthesis, polymer complex synthesis, and protein complex body synthesis. In the genes differentially expressed in L3vsL1, there were more downregulated genes than upregulated genes in most enriched items.

**Figure 5:**
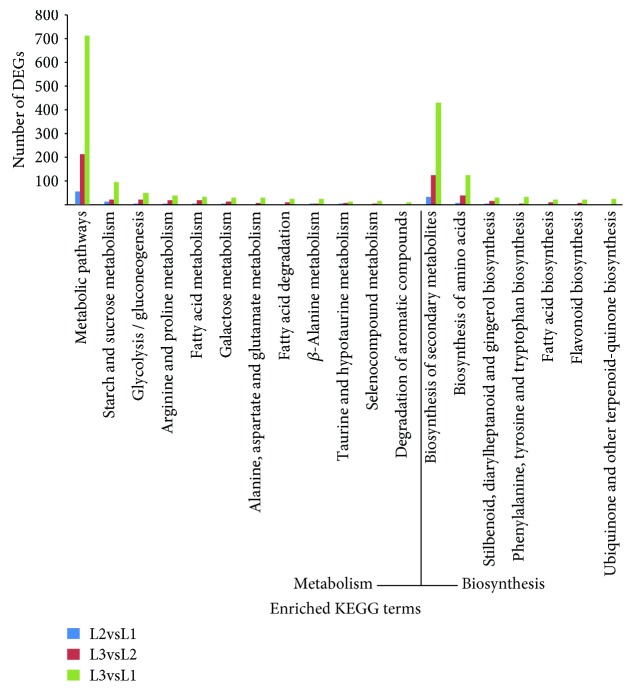
KEGG enrichment analysis of differentially expressed genes among different samples.

*(3) KEGG Metabolic Pathway Enrichment Analysis*. KEGG, an online database that contains information on genomes, enzymatic pathways, and biochemical substances, can be used to further study the complex behavior of genes [21]. The primary metabolic pathways and signal transduction pathways related to the DEGs of the walnut kernel during different developmental stages were identified by a KEGG enrichment analysis (Figure 5). The results showed that 307 genes that were differentially expressed in L2vsL1 were annotated to 77 metabolic pathways in the KEGG database, 1094 genes that were differentially expressed in L3vsL2 were annotated to 110 metabolic pathways, and 3717 genes that were differentially expressed in L3vsL1 were annotated to 121 metabolic pathways. The pathways enriched by more DEGs were primarily starch and sucrose metabolism, glycolysis/gluconeogenesis, amino acid metabolism, fatty acid metabolism, and secondary metabolite and amino acid synthesis. Among these pathways, the number of different genes expressed in different pathways varied, and the difference in genes of L3vsL1 in all pathways was greater than L3vsL2 and L2vsL1, indicating that the physiological processes also changed greatly with the development of the fruit.

*(4) Identification of Fat Metabolism-Related Genes*. The primary purpose of this study was to discover the mechanisms underlying walnut fat synthesis and to identify the key genes that control walnut fat synthesis. This analysis of the expression levels genes related to kernel fat biosynthesis at the 60 DAF, 90 DAF, and 120 DAF revealed certain genes that regulate the synthesis of walnut fat.

Among the differentially expressed genes annotated by KEGG, there were many genes related to fatty acid extension, unsaturated fatty acid biosynthesis, fatty acid metabolism, fatty acid degradation, fatty acid biosynthesis, and glycerolipid metabolism (Table 5).

Changes in the expression of these genes were likely the basis for the observed differences in fat content and fatty acid composition of the walnut kernel at various developmental stages.

The site of the initial synthesis of plant fatty acids is the plastid, and the synthesis is catalyzed by fatty acid synthase. Pyruvate dehydrogenase (PHD) generates acetyl coenzyme A (acetyl-CoA), which is the raw material for fatty acid synthesis. The first step of fatty acid biosynthesis is the conversion of acetyl-CoA to malonyl-CoA, catalyzed by acetyl coenzyme A carboxylase (ACC). Malonyl groups are condensed with acetyl-CoA, catalyzed by acyltransferase (ACP), followed by the sequential addition of 2 carbon units to extend the fatty acid chain [22]. A total of 23 differentially expressed genes involved in fatty acid synthesis were identified in this study, including 7 long-chain acyl-CoA synthetases (LACS), ACC1, 4 beta-ketoacyl-ACP synthases (KASs), FAB1, SSI2, FATB, FTM1, and MOD1.

Two important enzymes in the fat synthesis pathway are acetyl-CoA carboxylase and LACS. The expression of ACC1 was upregulated from L1 to L3 (Table 6). LACS plays an important role in lipid metabolism. Whether the reaction is anabolic or catabolic, the essential substrate, fatty acyl-CoA, is catalyzed by LACS. In this study, the expression of LACS1 was found to be upregulated from L1 to L3; the expression of LACS7 was not significantly different in L1 and L2 and was upregulated from L2 to L3. The expression of LACS9 was upregulated from L1 to L3 and was not significantly different between L2 and L1 or L3. KAS, which is an acyl carrier, catalyzes the condensation reaction of fatty acid synthesis [22]. In this study, a total of 4 KAS-related genes were detected, and their expression levels were downregulated from L1 to L3.

The content of unsaturated fatty acids in walnut fat is greater than 60%. In this experiment, 14 differentially expressed genes related to unsaturated fatty acid synthesis were found, including 5 desaturases, 3 thiolases, 2 dehydratases, 1 reductase, 1 oxidase, 1 protease, and 1 phosphatase. The expression levels of 2 sulfur lyases were upregulated, and 1 sulfur lyase was downregulated. Of the 5 desaturases, only FAD7 was upregulated. The expression levels of 2 PAS2 genes were upregulated, and the expression of Rossmann-fold NAD(P)-binding domain-containing protein was upregulated from L1 to L 2.

### 3.3. Validation of DEG Expression by qRT-PCR

**Figure 6:**
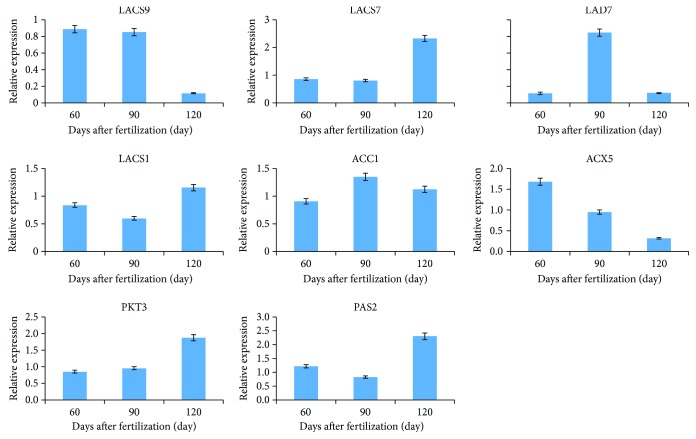
Expression analysis of fatty acid synthesis candidate genes in walnut kernels at different developmental stages.

To validate the expression of the DEGs obtained from RNA-Seq, 8 DEGs were selected for qRT-PCR, including LACS1, LACS7, LACS9, FAD7, ACX5, PKT3, PAS2, and ACC1. The expression trends of 8 DEGs were different; LACS1, LACS7, PKT3, and ACC1 genes were upregulated from 60 to 120 days after fertilization, while ACX5 and LACS9 were gradually downregulated (Figure 6). FAD7 upregulated rapidly at 90 days after fertilization and gradually downregulated after 120 DAF. Correlation analysis (Table 7) showed that the correlation coefficients of 8 genes related to the walnut lipid synthesis between expression and transcriptome sequencing were above 0.76, suggesting that our transcriptome analysis was accurate and reliable.

## 4. Discussion

The walnut kernel was found to be rich in unsaturated fatty acids. In this experiment, the fat content and fatty acid composition of the precocious Lvling walnut kernel were determined at various developmental stages. The results showed that the walnut kernel began to solidify at 50 DAF and that the fat content rapidly accumulated between 60 and 90 DAF, reaching a peak at 90 DAF and then decreasing. At 130 DAF, the fruit is mature and tends to stop growing. However, the trend of fatty acid composition was inconsistent with fat content, and this result was consistent with that of the study reported by Hu et al. [23]. The content of saturated fatty acids in the walnuts gradually decreased with the development of the fruit, whereas the content of unsaturated fatty acids exhibited an upward trend. This accumulation of fatty acid was similar to that of woody oil plants such as *Symplocos paniculata* [15] and *Swida wilsoniana* [24, 25]. Based upon the analysis of the fruit fat, the three developmental stages selected for further transcriptome analysis were 60, 90, and 120 DAF.

In this study, the transcriptome of the fruit at various developmental stages was sequenced using the Illumina technique, and the functions of the assembled unigenes were annotated. In addition, the differentially expressed genes related to fat synthesis were screened using the DESeq method. Certain key genes were also analyzed, and these genes included genes involved in the synthesis of acetyl-CoA and genes related to fatty acid synthesis. This study provides a theoretical basis for walnut fat synthesis. From the abovementioned analysis of genes encoding key enzymes and genes involved in the regulation process in the walnut fatty acid biosynthetic pathway, it was found that with gradual maturation, the fat content of the walnut kernel increased. The high expression of LACS and ACCase accelerates fatty acid biosynthesis. Thus, it can be concluded that LACS and ACCase are key targets for the gene regulation of the fat content in walnuts.

Acetyl-CoA carboxylase catalyzes the conversion of acetyl-CoA into malonyl-CoA, which constitutes the initial reaction in fatty acid biosynthesis [26]. Therefore, ACCase is a key enzyme in the process of fat synthesis and is a major rate-limiting enzyme [27, 28]. In many organisms, ACCases in plastids and in the cytosol catalyze the conversion of acetyl-CoA conversion into malonyl-CoA, but the function of ACCase in plants is not exactly the same. Malonyl-CoA generated by the ACCase in plastids is used for the de novo biosynthesis of fatty acids, whereas malonyl-CoA generated by ACCase in the cytosol is used for fatty acid extension, flavonoid formation, and the synthesis of many other secondary metabolites. The seed-specific overexpression or constitutive overexpression of ACC1, an *Arabidopsis* homozygous ACCase gene, increases the fatty acid content in *Arabidopsis* and the potato [29, 30]. In this study, ACC1, a differentially expressed ACCase gene, was found to be upregulated from stage 1 to stage 2, indicating that ACC1 is a key enzyme involved in walnut fat synthesis. Long-chain acyl-CoA synthase (LACS, EC 6.2.1) plays an important role in lipid metabolism; whether the reaction is anabolic or catabolic, the necessary substrate, fatty acyl-CoA, is catalyzed by LACS. Physiological processes in plants, such as fatty acid chain extension and desaturation modification, transport of fatty acids, and the generation of other lipid derivatives of fatty acids, are all dependent on LACS [31]. The LACS protein family in *Arabidopsis thaliana* contains LACS1–LACS9. LACS2 and LACS9 have been reported to play important roles in lipid synthesis and accumulation [32, 33]. In this study, LACS1, LACS2, LACS4, LACS7, and LACS9 were found to be differentially expressed in the various stages of walnut development. For example, LACS1, LACS7, and LACS9 were upregulated from stage 1 to stage 3, indicating that LACS played a key role in walnut kernel fatty acid synthesis.

In this study, the differentially expressed genes related to unsaturated fatty acid synthesis were also analyzed, and 14 differentially expressed genes were found. Fatty acid desaturases exist in all plants and can be classified into the following three groups according to their catalytic substrates [34]: (1) acyl-carrier protein desaturases, which are present in the plant cytoplasmic matrix, introduce double bonds into acyl carbon chains that bind to ACP; (2) acyl-CoA desaturases, which are present in the endoplasmic reticulum of plant cells, introduce double bonds into the acyl of acyl-CoA; and (3) acyl-lipid desaturases, which are present in the plasma membrane of plant cells, introduce double bonds into the acyl carbon chains of glycerolipids and glycolipids. In this study, 5 fatty acid desaturase-encoding genes were found, including 3 acyl-lipid desaturase genes (FAD2, FAD7) and 2 acyl-ACP desaturase genes.

The acyl-lipid desaturase catalyzes the fatty acid carbon chains binding with glycerol to form double bonds. The acyl-lipid desaturases screened in this study include 2 *ω*-6 fatty acid desaturases (FAD2) and 1 *ω*-3 fatty acid desaturase (FAD7). The *ω*-6 fatty acid desaturases introduce a double bond at the *ω*-6 position of the mono-olefm fatty acid carbon chain of glycerol to form the diene fatty acid carbon chain [35], whereas the *ω*-3 fatty acid desaturase catalyzes the biosynthesis of octadecatrienoic acid and hexadecatrienoic acid, forming the third double bond in a polyunsaturated fatty acid. *α*-Linolenic acid, which is present in walnut fat, is a polyunsaturated fatty acids with 3 double bonds, and FAD7 is most likely the key enzyme that catalyzes the synthesis of *α*-linolenic acid.

Many studies indicate that differences in the gene sequence could also influence the fat accumulation in walnut fruits. In this study, the genes related to walnut kernel fatty acid synthesis were screened, and their expression patterns during the lipid synthesis phase were analyzed. However, the differences in the sequence of genes affecting walnut fat content were not discussed in this paper. So, this study was the foundation for uncovering the mechanism underlying walnut kernel fatty acid formation. The mechanism of walnut oil synthesis still needs further study. We anticipate that future research based upon this study will reveal the entire mechanism underlying the synthesis of walnut fat.

## Figures and Tables

**Table 1:** qRT-PCR validation primers.

Gene	Primer sequence (5′ to 3′)	Tm (°C)
*β*-Actin	CTCTTCCAGCCATCCATGATCG	57.63
	CCACTGAGGACAATATTGCCAT	55.16
LACS7	CAGGAGTTGAGGTTGTGACATATTC	56.05
	AGAGCAGCCATATCATCCATTAGTT	56.28
LACS1	CAGGTTAGGTGGTCGGATTAGACT	57.86
	TGATGGCTCCTCAAGTGGATTGT	58.43
ACC1	GAGAATTGCTGAAGAGTCGCTGAT	57.73
	TGTTGGCTCCACCTTGCTTAGA	58.56
FAD7	ACTTCAACTCTGTGCTGGTCTCT	58.07
	CCTCCTCGTAGATAACTCCATTCCT	57.29
LACS9	CGACTTACCACTTCCAGCCGATA	58.66
	ACTTCCAACAGCAGCAATCACATT	58.22
ACX5	TGATGAGGGTTTCACAGGTTACAAG	57.32
	AAGCATAAGCAGAAGCCAGCAA	57.86
PKT3	GCAGGATGGCGGCATTCTATG	58.74
	AGAGGCAGTAGCAGCAGCAG	59.29
PAS2	CGTCTTCTTCGGATGGCTTCA	57.33
	CAGTGTTGCTGAGATCGGAGAT	56.92

**Table 2:** Dynamic changes of fatty acid content in walnut kernel fat (%).

Types of fatty acids		Days after fertilization/day							
		60	70	80	90	100	110	120	130
Unsaturated fatty acid	Linoleic acid (C18 : 2)	60.86 ± 2.01f	66.87 ± 2.10c	68.36 ± 2.56b	69.66 ± 2.03a	64.99 ± 2.34d	64.53 ± 2.01d	63.29 ± 2.35e	64.34 ± 1.32d
	Oleic acid (C18 : 1)	6.67 ± 0.13 g	8.37 ± 0.03f	12.29 ± 0.12de	12.87 ± 0.15d	18.92 ± 0.21bc	19.53 ± 0.45ab	20.15A ± 0.20a	19.28 ± 0.13b
	*α*-Linolenic acid (C18 : 3)	12.02 ± 0.08ab	12.22 ± 0.12a	9.3 ± 0.05c	8.54 ± 0.03d	7.38 ± 0.04e	6.88 ± 0.10 g	7.36 ± 0.06ef	6.93 ± 0.04 fg
	Palmitoleic acid (C16 : 1)	0.25 ± 0.02a	0.15 ± 0.03b	0.13 ± 0.001b	0.06 ± 0.001c	0.06 ± 0.001c	0.06 ± 0.002c	0.07 ± 0.001c	0.06 ± 0.002c
	*all*-*cis*-11,14-Eicosadienoic acid (C20 : 2)	0.07 ± 0.01a	0.08 ± 0.001a	0.03 ± 0.001b	0.04 ± 0.001b	0.02 ± 0.001bc	0.03 ± 0.001b	0.03 ± 0.001b	0.02 ± 0.001bc
	*cis*-11-Eicosenoic acid (C20 : 1)	0.40 ± 0.003a	0.26 ± 0.002b	0.19 ± 0.001c	0.19 ± 0.001c	0.19 ± 0.001c	0.17 ± 0.001c	0.17 ± 0.001c	0.18 ± 0.001c

Saturated fatty acid	Margaric acid (17 : 0)	0.12 ± 0.01	0.06 ± 0.001	0.07 ± 0.001	0.07 ± 0.001	0.06 ± 0.001	0.07 ± 0.001	0.07 ± 0.001	0.07 ± 0.001
	Behenic acid (22 : 0)	0.34 ± 0.003a	0.10 ± 0.001b	0.02 ± 0.001b	0.02 ± 0.001b	0.03 ± 0.001b	0.01 ± 0.001b	0.03 ± 0.001b	0.02 ± 0.001b
	Lauric acid (12 : 0)	0.06 ± 0.001a	0.01 ± 0.001b	0b	0.02 ± 0.001b	0.02 ± 0.001b	0.02 ± 0.001b	0.01 ± 0.001b	0b
	Pentadecylic acid (15 : 0)	0.25 ± 0.002a	0.08 ± 0.001b	0.04 ± 0.001c	0.02 ± 0.001c	0.02 ± 0.001c	0.01 ± 0.001c	0.02 ± 0.001c	0.02 ± 0.001c
	Myristic acid (C14 : 0)	0.13 ± 0.005a	0.07 ± 0.001b	0.06 ± 0.001b	0.03 ± 0.001c	0.02 ± 0.001c	0.02 ± 0.001c	0.02 ± 0.001c	0.01 ± 0.001c
	Palmitic acid (C16 : 0)	17.45 ± 0.01a	10.06 ± 0.001b	7.57 ± 0.02c	5.93 ± 0.04ef	5.35 ± 0.03 fg	5.85 ± 0.01ef	6.06 ± 0.02de	6.46 ± 0.03d
	Stearic acid (C18 : 0)	1.29 ± 0.003e	1.59 ± 0.001d	1.90 ± 0.001 cd	2.5 ± 0.001bc	2.87 ± 0.005a	2.75 ± 0.007a	2.65 ± 0.002ab	2.55 ± 0.008bc
	Arachidic acid (20 : 0)	0.09 ± 0.002a	0.08 ± 0.001a	0.05 ± 0.001b	0.05 ± 0.001b	0.07 ± 0.001a	0.07 ± 0.001a	0.07 ± 0.001a	0.06 ± 0.001ab

Note: values represent the mean ± SE. Means within each line followed by the same letter are not significantly different at *P* < 0.05 according to Duncan's multiple range test.

**Table 3:** Data statistics of transcriptome sequencing.

Sample name	Number of clean reads	Clean bases (G)	Unique mapped reads	Q20 (%)	Q30 (%)	GC content
L1–1	56,786,156	8.52	46,399,939	97.06	92.63	47.34
L1–2	47,864,764	7.18	39,778,196	97.04	92.56	46.74
L1–3	48,659,022	7.3	40,863,104	96.93	92.35	46.49
L2–1	51,398,780	7.71	39,559,096	96.91	92.24	49.13
L2–2	52,897,882	7.93	43,079,514	96.94	92.34	47.63
L2–3	50,390,996	7.56	39,187,890	96.75	91.88	49.64
L3–1	50,680,344	7.6	41,211,813	96.78	92.01	48.31
L3–2	46,300,696	6.95	37,672,468	97.01	92.46	48.35
L3–3	49,505,362	7.43	40,171,554	96.97	92.44	48.01
Total	454,484,002	68.18				

**Table 4:** KEGG analysis of common differentially expressed genes.

KEGG pathway	Pathway ID	Input number
Protein processing in endoplasmic reticulum	ath04141	15
Taurine and hypotaurine metabolism	ath00430	3
Starch and sucrose metabolism	ath00500	12
Endocytosis	ath04144	8
Photosynthesis-antenna proteins	ath00196	3
Plant-pathogen interaction	ath04626	9
Stilbenoid, diarylheptanoid, and gingerol biosynthesis	ath00945	4
Flavonoid biosynthesis	ath00941	2
Carbon fixation in photosynthetic organisms	ath00710	4
SNARE interactions in vesicular transport	ath04130	3
Phenylpropanoid biosynthesis	ath00940	7
Biosynthesis of secondary metabolites	ath01110	33
RNA degradation	ath03018	4
Phenylalanine metabolism	ath00360	4
Spliceosome	ath03040	6
Metabolic pathways	ath01100	54
Glycolysis/gluconeogenesis	ath00010	3
Carbon metabolism	ath01200	6
Biosynthesis of amino acids	ath01230	6

**Table 5:** KEGG analysis of differentially expressed genes in lipid metabolism.

Term	Database ID	Background number	Input number		
			L2vsL1	L3vsL2	L3vsL1
Fatty acid metabolism	ath01212	73	4	17	32
Biosynthesis of unsaturated fatty acids	ath01040	36	2	7	11
Fatty acid biosynthesis	ath00061	40	2	9	20
Fatty acid degradation	ath00071	40	2	10	22
Fatty acid elongation	ath00062	31	1	4	13
Glycerophospholipid metabolism	ath00564	83	1	13	31
Glycerolipid metabolism	ath00561	52	0	5	22

**Table 6:** Differentially expressed genes in fatty acid synthesis.

Gene ID	Gene symbol	Annotation	L2vsL1	L3vsL2	L3vsL1
WALNUT_00023412	LACS1	Long-chain acyl-CoA synthetase 1		D	D
WALNUT_00006224	LACS1	Long-chain acyl-CoA synthetase 1			U
WALNUT_00028238	LACS2	Long-chain acyl-CoA synthetase 2		U	D
WALNUT_00012724	LACS4	Long-chain acyl-CoA synthetase 4	D		D
WALNUT_00003419	LACS7	Long-chain acyl-CoA synthetase 7		U	U
WALNUT_00018437	LACS7	Long-chain acyl-CoA synthetase 7		D	D
WALNUT_00022757	LACS9	Long-chain acyl-CoA synthetase 9			U
WALNUT_00011609	FAB1	3-Oxoacyl-[acyl-carrier-protein] synthase		D	
WALNUT_00012795	SSI2	Acyl-[acyl-carrier-protein] desaturase		D	D
WALNUT_00014166	FATB	Fatty acyl-ACP thioesterases		D	
WALNUT_00019717	FTM1	Stearoyl-ACP desaturase		D	U
WALNUT_00021234	CAC3	Acetyl coenzyme A carboxylase carboxyl transferase subunit alpha		D	D
WALNUT_00022861	FATA	FatA acyl-ACP thioesterase		D	D
WALNUT_00002636	MOD1	Enoyl-[acyl-carrier protein] reductase I		D	D
WALNUT_00009311	MOD1	Enoyl-[acyl-carrier-protein] reductase (NADH)		D	D
WALNUT_00012185	ACC1	Homomeric acetyl-CoA carboxylase (Hom-ACCase)	U		U
WALNUT_00024655	KAS_III	3-Ketoacyl-[acyl-carrier-protein] synthase III			D
WALNUT_00025417	KAS_I	Beta-ketoacyl-[acyl-carrier-protein] synthase I		D	D
WALNUT_00028064	KAS_I	Beta-ketoacyl-[acyl-carrier-protein] synthase I		D	D
Novel01692	KAS_I	Beta-ketoacyl-[acyl-carrier-protein] synthase I		D	D
WALNUT_00011323		3-Hydroxyacyl-[acyl-carrier-protein] dehydratase		D	D
WALNUT_00003181		NAD(P)-binding Rossmann-fold superfamily protein		D	D
WALNUT_00018045		Short-chain type dehydrogenase		D	
WALNUT_00021629	PKT3	3-Ketoacyl-CoA thiolase 2			U
WALNUT_00026180	PKT3	3-Ketoacyl-CoA thiolase 2		U	
WALNUT_00009879	PKT3	3-Ketoacyl-CoA thiolase 2			D
WALNUT_00003181		3-Oxoacyl-[acyl-carrier-protein] reductase		D	D
WALNUT_00011902	FAD2	Omega-6 fatty acid desaturase *ω*-6	D		D
WALNUT_00002632	FAD2	Omega-6 fatty acid desaturase *ω*-6		D	
WALNUT_00015387	FAD7	Fatty acid desaturase 7	U		U
WALNUT_00018114	PAS2	Very-long-chain (3R)-3-hydroxyacyl-[acyl-carrier protein] dehydratase			U
WALNUT_00026237	PAS2	Very-long-chain (3R)-3-hydroxyacyl-[acyl-carrier protein] dehydratase		U	U
WALNUT_00018045		Rossmann-fold NAD(P)-binding domain-containing protein	U	D	
WALNUT_00019717		Acyl-[acyl-carrier-protein] desaturase		D	D
WALNUT_00020859		Protein-tyrosine phosphatase			D
WALNUT_00008997	ACX5	Acyl-CoA oxidase 5		D	D
WALNUT_00012795	SSI2	Acyl-[acyl-carrier-protein] desaturase	D		D

Note: U: significantly upregulated; D: significantly downregulated.

**Table 7:** Correlation coefficients of candidate genes related to the walnut lipid synthesis between expression and transcriptome sequencing.

Gene ID	Enzyme names	Correlation coefficient
WALNUT_00003419	LACS7	0.92
WALNUT_00006224	LACS1	0.96
WALNUT_00008997	ACX5	0.99
WALNUT_00012185	ACC1	0.88
WALNUT_00015387	FAD7	0.96
WALNUT_00021629	PKT3	0.98
WALNUT_00022757	LACS9	0.76
WALNUT_00026237	PAS2	0.97

## Data Availability

The FASTA data used to support the findings of this study were supplied by Hebei Agricultural University under license and so cannot be made freely available. Requests for access to these data should be made to Guohui Qi, bdqgh@163.com.
